# *In Vitro* Antibacterial Potential of *Pinus nigra*-*Thymus serpyllum* Essential Oil and Antibiotic Combinations

**DOI:** 10.1021/acsomega.5c07183

**Published:** 2025-12-09

**Authors:** Sümeyye Elif Kahya, Ayşe Esra Karadağ, Betül Demirci, Fatih Demirci

**Affiliations:** † Department of Pharmacognosy, School of Pharmacy, 218502 Istanbul Medipol University, 34820 Beykoz, Istanbul, Türkiye; ‡ Department of Pharmacognosy, Faculty of Pharmacy, 52944 Anadolu University, 26210 Eskişehir, Türkiye

## Abstract

Synergistic interactions have been recognized and used for centuries, with various healing traditions using herbal combination treatments to enhance their effects. In the recent years, essential oil combinations were used effectively. *Pinus nigra* JF Arnold, and *Thymus serpyllum* L. essential oils, which are mainly used in colds and respiratory diseases, were examined for the efficacy, individually and in combination against 12 different human pathogens. The antimicrobial effects were evaluated using the *in vitro* microdilution method and the checkerboard assay to assess potential synergistic interactions. Herein comparative inhibitions by *T. serpyllum* and *P. nigra* oils with the combination of ciprofloxacin, amoxicillin, and tetracycline antibiotics were tested, respectively. The major components of the essential oils were also evaluated in combination. The quality of the commercial essential oils were analyzed by GC/MS and GC/FID for quality confirmation. The main components for *P. nigra* were α-pinene (73.8%), *trans*-verbenol (4.4%), and α-pinene oxide (2%), respectively, whereas for *T. serpyllum*, the primary components were geraniol (19.3%), *p*-cymene (12.9%), α-thujone (6.6%), linalool (6.4%), and carvacrol (5.7%), respectively. The microbiological evaluations resulted in a synergistic effect of *T. serpyllum + P. nigra* oil combination against *Enterococcus faecalis* (FICI: 0.187), and *Staphylococcus epidermidis* (FICI: 0.375). The combination of *P. nigra* and amoxicillin also resulted in a synergistic effect against the *Streptococcus mitis* (FICI: 0.185) strain. In addition, the combination of the main components of the essential oils were evaluated against all microorganisms in the panel and resulted in independent activity. Using the *in vitro* MTT assay, the synergistic combinations resulted in no toxicity.

## Graphical abstract

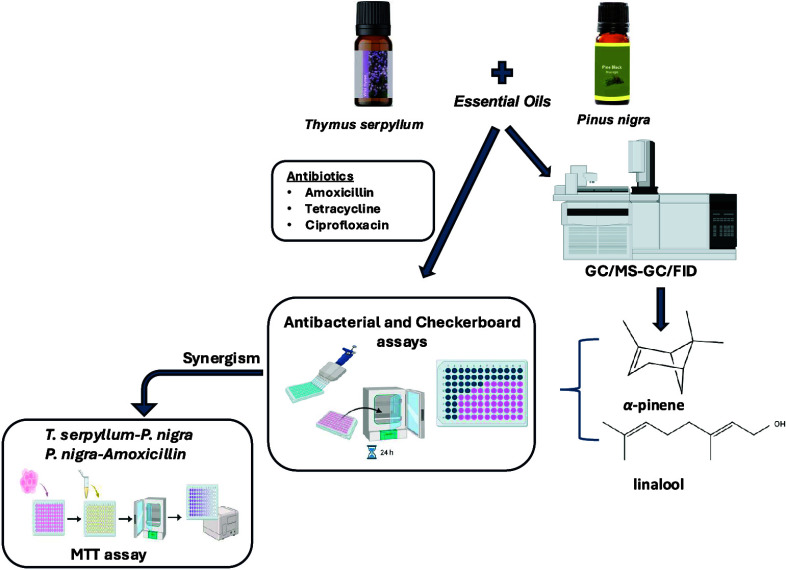

## 1 Introduction

*Thymus serpyllum* L. (Lamiaceae) is commonly known as a ‘wild thyme’, and is originally from Eastern Mediterranean regions, where also a monograph of the dried flowering aerial parts in the European Pharmacopoeia exists as Serpylli herba, which is widely used in Europe.^1^ Carvacrol was reported as the main component in *T. serpyllum*, with variable amounts of thymol, linalool, linalyl acetate, borneol, 1,8-cineole, germacrene D, *p*-cymene, γ-terpinene, myrcene, and β-caryophyllene, respectively. The content varies significantly based on the species, genetic variation, developmental stage, origin, and cultivation conditions, so its chemotype diversity occurs.^2,3^ Its preparations are traditionally used for treating colds, rheumatism, sciatica, and its stimulating, disinfectant, and immune-stimulating effects.^4,5^

*Pinus nigra* JF Arnold (Black pine) is a diverse species which belongs to the Pinaceae family, present throughout the Mediterranean region.^6^ The species is divided into seven subspecies: *Pinus nigra* subsp. *dalmatica* (Vis.) Franco, *P. nigra* subsp. *laricio* Palib. ex Maire, *P. nigra* subsp. *nigra*, *P. nigra* subsp. *pallasiana* (Lamb.) Holmboe, *P. nigra* subsp. *salzmannii* (Dunal) Franco, *P. nigra* f. *seneriana* (Saatçioglu) Kandemir & Mataraci, and *P. nigra* var. *yaltirikiana* Alptekin.^7^ Black pine is recognized for its genetic, morphological, phenotypic, and biochemical diversity. The chemical composition of essential oils varies among different origins and subspecies, with the abundance of monoterpenes such as α-pinene, β-pinene, limonene, δ-3-carene, and β-phellandrene among others.^8,9^ Biological activities such as antibacterial, antifungal, and antioxidant are reported within traditional medicine.^10^

Antimicrobial resistance can be classified as one of the clinical and economical devastating impacts globally. Pathogens such as *Staphylococcus aureus* and *Pseudomonas aeruginosa* are frequently listed as priority pathogens by the World Health Organization, because they rank as leading causes for infections acquired in communities along with hospitals worldwide.^11,12^ According to the One Health approach, the spread of resistant strains constitutes a significant threat to human, animal, and environmental health. Consequently, new research for new innovative antimicrobial agents such as essential oils and combinations withdraw attention as a sustainable strategy.^13^

The combination of conventional antibiotics with EOs enhances their effectiveness, positioning EOs as promising alternatives to synthetic antimicrobial agents for combating resilient infections, where recent studies demonstrated synergistic activity in specific combinations.^14,15^ Synergistic effects have significant advantages, such as the drug combination exerting a more substantial impact than the sum of the drugs’ effect, lower toxicity, and reduced side effects.^16^

In this present study, to achieve synergistic antimicrobial activity *T. serpyllum* and *P. nigra* essential oil combinations were systematically evaluated. The checkerboard method was used to evaluate the binary combinations of the essential oils and their individual combinations with tetracycline, amoxicillin, and ciprofloxacin, respectively. In addition, based on the GC/MS and GC/FID verification results, the combinations of the major components geraniol and α-pinene were evaluated against pathogenic microorganisms. To the best our knowledge, the combination of *T. serpyllum* and *P. nigra* essential oils, tetracycline, amoxicillin, ciprofloxacin, major components geraniol and α-pinene, respectively, were evaluated for the first time. Also, the active combinations were further assessed for their potential toxicity using the *in vitro* MTT assay.

## 2 Result and Discussion

### 2.1 Phytochemical Analysis

The detailed chromatographic analyses represent the relative percentages (%) calculated from FID data which are listed in Table 1. GC-FID and GC/MS analyses of the commercial essential oils verified 20 and 46 compounds, representing 92% and 97.2% of the essential oils, respectively. The main compounds for *P. nigra* oil were characterized as α-pinene (73.8%), *trans*-verbenol (4.4%), α-pinene oxide (2%), camphene (1.7%), limonene (1.7%), verbenone (1.6%), and β-pinene, respectively. The main compounds for *T. serpyllum* were geraniol (19.3%), *p*-cymene (12.9%), α-thujone (6.6%), linalool (6.4%), carvacrol (5.7%), geranyl acetate (5.6%), geranyl formate (4.3%), and camphor (3.7%), respectively. *T. serpyllum* and *P. nigra* essential oil (see Suppl. For Chromatograms in Figure S1 and S2).

**Table 1:** Essential Oil Compositions of *T. serpyllum* and *P. nigra* Essential Oils^a^

RRI	Component	PN (%)	TS (%)
1014	Tricyclene	0.1	0.1
1032	α-Pinene	73.8	1.2
1035	α-Thujene	-	0.4
1076	Camphene	1.7	1.7
1100	Undecane	-	-
1118	β-Pinene	1.4	0.4
1132	Sabinene	-	0.1
1135	Thuja-2,4(10)-diene	0.1	-
1174	Myrcene	-	0.7
1188	α-Terpinene	-	0.6
1203	Limonene	1.7	-
1213	1,8-Cineole	-	1.3
1255	γ-Terpinene	-	1.8
1265	3-Octanone	-	0.3
1280	*p*-Cymene	0.1	12.9
1298	Terpinolene	-	0.2
1384	α-Pinene oxide	2.0	-
1437	α-Thujone	-	6.6
1439	γ-Campholene aldehyde	0.1	-
1451	β-Thujone	-	2.2
1452	1-Octen-3-ol	-	0.1
1452	*α-p*-Dimethyl styrene	-	0.1
1499	α-Campholene aldehyde	0.1	-
1532	Camphor	-	3.7
1535	β-Bourbonene	0.3	-
1536	Pinocamphone	0.1	-
1553	Linalool	-	6.4
1565	Linalyl acetate	-	0.5
1571	*trans*-*p*-Menth-2-en-1-ol	-	0.1
1586	Pinocarvone	0.2	-
1590	Bornyl acetate	-	0.5
1604	Thymol methyl ether	-	0.5
1611	Terpinen-4-ol	-	2.5
1612	β-Caryophyllene	-	2.3
1614	Carvacrol methyl ether	-	1.9
1638	*cis*-*p*-Menth-2-en-1-ol	-	0.1
1648	Myrtenal	0.4	-
1662	Pulegone	-	0.2
1670	*trans*-Pinocarveol	2.0	-
1683	*trans*-Verbenol	4.4	-
1687	α-Humulene	-	1.6
1694	Neral	-	0.2
1706	α-Terpineol	-	0.9
1709	α-Terpinyl acetate	-	0.5
1715	Geranyl formate	-	4.3
1719	Borneol	-	0.7
1725	Verbenone	1.6	-
1726	Germacrene D	-	0.2
1740	Geranial	-	1.0
1741	β-Bisabolene	-	0.1
1751	Carvone	0.2	-
1784	*(E)-α*-Bisabolene	-	1.0
1795	Geranyl acetate	-	5.6
1804	Myrtenol	0.8	-
1808	Nerol	-	0.5
1845	*trans*-Carveol	0.1	-
1857	Geraniol	-	19.3
2008	Caryophyllene oxide	-	0.5
2071	Humulene epoxide II	-	0.4
2104	Viridiflorol	-	2.0
2198	Thymol	-	3.3
2239	Carvacrol	-	5.7
	**Total**	**92.0**	**97.2**

a PN: *Pinus nigra oil*, TS: Thymus serpyllum oil, RRI: Relative retention indices calculated against *n*-alkanes,% relative percentages, (−) not detected.

In a previous study, *T. serpyllum* essential oil reported 10 different chemotypes, namely: carvacrol, 1,8-cineole/caryophyllene oxide, *p*-cymene, geraniol, linalool, *trans*-nerolidol, γ-terpinene, α-terpinol acetate, linalyl acetate, and thymol.^17–19^ Among all, the most common chemotypes of *T. serpyllum* were suggested as the carvacrol and thymol type.^3,20^ Paaver et al.^19^ observed four chemotypes (*trans*-nerolidol, caryophyllene oxide, myrcene, and borneol) growing in Estonia. Another study reported that *T. serpyllum* chemotypes are thymol, caryophyllene oxide, τ-cadinol, β-cubebene, geraniol, geranyl isobutyrate, and 1,8-cineole from Hungary. In this present study, the utilized commercial *T. serpyllum* essential oil can be classified as geraniol chemotype, in agreement with previous reports in varying concentrations.^17,21^

The major compound of commercial *P. nigra* essential oil was reported as α-pinene, according to phytochemical analyses.^22,23^ . Regarding the *Pinus* essential oils in previous reports; α-pinene (44.98%), β-pinene (14.27%), germacrene D (9.63%), limonene (6.93%), and β-caryophyllene (5.58%), respectively was identified in the *P. nigra* needle oil.^22^ In another study, the main components of pine essential oil were reported as α-pinene (45.93%), germacrene D (27.50%), (*E*)-caryophyllene (8.13%), and β-pinene (6.90%).^23^

When compared with literature data, the chemical compositions and major compounds showed consistency to previous studies.

### 2.2 Broth Microdilution Assay

The antimicrobial activity of essential oil of *T. serpyllum* and *P. nigra* was evaluated at a 1000 μg/mL concentration against the pathogenic microorganisms, as shown in Table 2. *P. nigra* essential oil was effective at relatively low concentrations against *B. cereus* (250 μg/mL) and *E. faecalis* (500 μg/mL). On the other hand, pine oil with MICs ranging from 1000 to >1000 μg/mL were moderate in activity. *T. serpyllum* oil was inhibitory against Gram-positive *S. aureus* and Gram-negative bacteria with MICs ranging from 500 to >1000 μg/mL.

**Table 2:** Comparative Antibacterial MIC (μg/mL) Data

Microorganisms	*T. serpyllum*	*P. nigra*	Geraniol	α-pinene	Amoxicillin	Tetracycline	Ciprofloxacin
*Bacillus cereus*	1000	250	5	>10	0.25	1	0.01
*Bacillus subtilis*	1000	1000	10	>10	0.5	0.250	0.25
*Enterococcus faecalis*	1000	500	1.25	>10	4	0.06	0.5
*Escherichia coli*	>1000	>1000	>10	>10	1	1	0.12
*Klebsiella pneumoniae*	>1000	>1000	>10	>10	0.5	8	0.01
*Moraxella catarrhalis*	1000	1000	10	>10	1	8	0.5
*Salmonella typhimurium*	>1000	>1000	>10	>10	8	8	0.03
*Staphylococcus aureus*	>1000	>1000	1.25	>10	0.5	8	0.25
*Staphylococcus aureus* BAA44	>1000	>1000	5	>10	4	8	1
*Staphylococcus epidermidis*	1000	1000	1.25	>10	1	8	0.01
*Streptococcus mitis*	>1000	>1000	10	>10	8	0.5	0.5
*Streptococcus mutans*	500	1000	>10	>10	1	0.5	0.03

The major compounds identified through phytochemical analysis were evaluated for their antimicrobial activity against the tested microorganisms. The minimum inhibitory concentration (MIC) of α-pinene for the microorganisms that were tested was determined to be 10 μg/mL. Geraniol exhibited MIC values of 1.25 μg/mL against *E. faecalis*, *S. aureus*, and *S. epidermidis*.

Essential oil of *T. serpyllum* showed potent inhibitory activity against *S. aureus* and *Candida tropicalis*.^24^ *T. serpyllum*, two major contents of carvacrol and thymol, and the antibacterial activity of both compounds on a broad spectrum of microorganisms are reported.^25–28^ Another study reported that *P. nigra* essential oil of antimicrobial activity low effectiveness against multiresistant bacterial strains isolated from humans.^29^ In a previous study published by Rosato et al.,^30^ pine oil found MIC values ranging from 0.5 to 4.1 mg/mL against Gram-negative and Gram-positive bacteria. According to a study by Demirci et al.^31^ essential oil obtained from *P. nigra* needles and cones was tested against human pathogens. The essential oil collected and obtained in April showed antimicrobial activity against *K. pneumoniae* and *E. coli*, while the essential oil collected and obtained in June was more effective against the tested microorganisms.

### 2.3 Checkerboard Assay

The outcomes of antimicrobial combination interactions are presented in Table 3. The fractional inhibition concentration index (FICI) values were calculated to evaluate the synergistic and additive activity. The results showed that the *T. serpyllum* and *P. nigra* essential oil combination provided synergistic effects against *E. faecalis* and *S. epidermidis*, and this combination of FIC index was 0.187 and 0.375, respectively. In the present study, the combination of *P. nigra* essential oil with amoxicillin resulted in a synergistic effect (FICI: 0.185) against *S. mitis*. The major components geraniol and α-pinene combinations whereas resulted as ‘‘independent’’ as an outcome against the tested pathogenic microorganisms as illustrated in Table 4.

**Table 3:** FICI Values of Combinations (EOs-EOs and EOs-Antimicrobial Agents)^a^

Microorganisms	Combinations	FICI	Results
*S. mutans*	TS-PN	0.515	Additive
	TS-AMX	1.500	Independent
	TS-TET	0.750	Additive
	TS-CIP	0.531	Additive
	PN-AMX	1.062	Independent
	PN-TET	2.000	Independent
	PN-CIP	0.515	Additive
*S. aureus* BAA44	TS-PN	2.000	Independent
	TS-AMX	1.000	Independent
	TS-TET	2.000	Independent
	TS-CIP	0.562	Additive
	PN-AMX	1.250	Independent
	PN-TET	2.000	Independent
	PN-CIP	1.015	Independent
*S. aureus*	TS-PN	2.000	Independent
	TS-AMX	1.015	Independent
	TS-TET	2.000	Independent
	TS-CIP	0.515	Additive
	PN-AMX	1.015	Independent
	PN-TET	1.015	Independent
	PN-CIP	0.530	Additive
*S. mitis*	TS-PN	2.000	Independent
	TS-AMX	0.500	Additive
	TS-TET	0.515	Additive
	TS-CIP	0.750	Additive
	PN-AMX	**0.185**	**Synergistic**
	PN-TET	2.000	Independent
	PN-CIP	1.015	Independent
*E. faecalis*	TS-PN	**0.187**	**Synergistic**
	TS-AMX	1.031	Independent
	TS-TET	0.506	Additive
	TS-CIP	0.531	Additive
	PN-AMX	0.560	Additive
	PN-TET	1.030	Independent
	PN-CIP	0.515	Additive
*M. catarrhalis*	TS-PN	1.500	Independent
	TS-AMX	1.015	Independent
	TS-TET	0.750	Additive
	TS-CIP	1.015	Independent
	PN-AMX	1.030	Independent
	PN-TET	1.500	Independent
	PN-CIP	0.515	Additive
*E. coli*	TS-PN	2.000	Independent
	TS-AMX	0.515	Additive
	TS-TET	1.015	Independent
	TS-CIP	1.031	Independent
	PN-AMX	0.530	Additive
	PN-TET	1.015	Independent
	PN-CIP	1.015	Independent
*K. pneumoniae*	TS-PN	2.000	Independent
	TS-AMX	2.000	Independent
	TS-TET	2.000	Independent
	TS-CIP	0.531	Additive
	PN-AMX	2.000	Independent
	PN-TET	2.000	Independent
	PN-CIP	0.530	Additive
*S. typhimurium*	TS-PN	2.000	Independent
	TS-AMX	0.625	Additive
	TS-TET	0.515	Additive
	TS-CIP	0.625	Additive
	PN-AMX	0.562	Additive
	PN-TET	1.015	Independent
	PN-CIP	0.515	Additive
*S. epidermidis*	TS-PN	**0.375**	**Synergistic**
	TS-AMX	1.250	Independent
	TS-TET	2.000	Independent
	TS-CIP	1.015	Independent
	PN-AMX	0.750	Additive
	PN-TET	2.000	Independent
	PN-CIP	1.015	Independent
*B. cereus*	TS-PN	0.500	Additive
	TS-AMX	1.015	Independent
	TS-TET	0.503	Additive
	TS-CIP	0.750	Additive
	PN-AMX	0.750	Additive
	PN-TET	0.506	Additive
	PN-CIP	0.560	Additive
*B. subtilis*	TS-PN	0.750	Additive
	TS-AMX	0.560	Additive
	TS-TET	1.015	Independent
	TS-CIP	1.031	Independent
	PN-AMX	0.560	Additive
	PN-TET	1.015	Independent
	PN-CIP	1.015	Independent

a **TS:** *T. serpyllum* oil; **PN:** *P. nigr*a oil; **AMX:** Amoxicillin; **CIP:** Ciprofloxacin; **TET:** Tetracycline.

**Table 4:** FICI Value Results of Geraniol + α-Pinene Combination

Microorganisms	FICI	Results
*S. mutans*	2.000	Independent
*S. aureus* BAA44	1.015	Independent
*S. aureus*	1.015	Independent
*S. mitis*	2.000	Independent
*E. faecalis*	1.015	Independent
*M. catarrhalis*	1.015	Independent
*E. coli*	2.000	Independent
*K. pneumoniae*	2.000	Independent
*S. typhimurium*	2.000	Independent
*S. epidermidis*	1.015	Independent
*B. cereus*	1.015	Independent
*B. subtilis*	1.015	Independent

This is the first data on the synergistic activity of *T. serpyllum + P. nigra* essential oil and their major components as well as selected antibiotic combinations against microbial pathogens, with promising outcomes. It is also important to highlight that the created combinations were evaluated against resistant bacterial pathogens. To the best of our knowledge, data on combination studies with *P. nigra* essential oil are scarce, supporting the novelty of the present study.

According to Karadağ et al.^15^ while the *T. serpyllum* and chlorhexidine combination showed an antagonisict effect against *S. mutans*, a partial synergism was observed in the combination tested against *S. aureus* pathogens. Ouedrhiri et al.^32^ reported the antimicrobial activity of combinations of *T. serpyllum* with other essential oils. The combination of *Origanum majorana* and *T. serpyllum* essential oils demonstrated a significant synergistic antibacterial activity against *S. aureus*. In contrast, the impact between these two essential oils against *E. coli* revealed an antagonistic result. Additionally, the combination of *Origanum compactum* and *T. serpyllum* oils demonstrated a notable synergistic effect against *S. aureus. Lavandula angustifolia* L. and *T. serpyllum* combination results highlighted a partial synergy effect against both strains. Also, Ouedrhiri et al.^33^ evaluated the combination of *T. serpyllum*, *Artemisia herba-alba*, and *Myrtus communis* essential oils in different concentrations. Ternary mixture combination consisting of *M. communis* (17.1%), *A. herba-alba* (39.6%), and *T. serpyllum* (43.1%) was reported to have potent inhibitory activity against *B. subtilis.* Salaria et al.^34^ reported the combination of *T. serpyllum* and different antibiotics (fluconazole and amphotericin B) against *Candida albicans*.

Combination studies of the major components were independently observed against all tested microorganisms. The mechanism of action for geraniol may be attributed to its ability to adhere to the cell membrane lipids of the microorganisms, whereas α-pinene exhibits an antimicrobial effect that disrupts cell wall integrity.^35,36^ Furthermore, combinations of geraniol and norfloxacin, chloramphenicol, and tetracycline were studied against several Gram-negative and Gram-positive strains. The geraniol-chloramphenicol, geraniol-norfloxacin, and geraniol-tetracycline combinations showed a synergism effect against *E. coli*.^37,38^ Another study showed that α-pinene damage cell membranes, causing ion leakage, which results in cell death.^39^

This present study evaluated various combinations of *T. serpyllum* and *P. nigra* oils, their major constituents geraniol-α-pinene, as well as the essential oil-antibiotics, respectively. A total of 3 synergistic combinations were identified. Numerous studies in the literature reported combinations involving the *T. serpyllum* essential oil with synergistic antimicrobial activity. *P. nigra* combination studies were tested for microbial activity for the first time in this study. Although *T. serpyllum* and *P. nigra* essential oils showed a synergistic effect against *S. epidermidis* and *E. faecalis*, the combinations of their major constituents represented independent effects against both pathogens. As per the observed results, the synergistic effect of essential oils may result from the oil composition rather than from the individual major components. Synergistic effects were also observed from the combination of *P. nigra* with antimicrobial agents, specifically with amoxicillin against *S. mitis*. For all the tested microorganisms, other combinations showed independent or additive effects. The independent effect is likely caused by one of the components within the combination acting on its own. An additive effect commonly occurs when two comparable antibiotics are combined to achieve the same level of activity, while minimizing the specific side effects associated with one. In addition to synergistic activity, partial synergy was observed in all combinations that exhibit additive effects.

### 2.4 Toxicity Assay

*In vitro* toxicity tests of the combinations with synergic effects were performed on the HEK293 cell line using the MTT method to evaluate selectivity. According to the FIC values, the combinations of *P. nigra-T. serpyllum* and *P. nigra*-amoxicillin were selected for the toxicity evaluation of the essential oils and amoxicillin alone. When the concentrations determined based on the FIC values, all combinations showed higher cell viability compared to the individual MIC values. Based on the data obtained from the cell culture toxicity, a reduction was observed in the combination studies.

According to Nikolić et al.^21^ the wild thyme oil, which contains thymol as its major constituent, showed relatively less *in vitro* cytotoxic activity against four human tumor cells. Deb et al.^40^ reported the *T. serpyllum* essential oil with antiproliferative activity against HL-60 cells (promyelocytic leukemia).

In this present study, designed combinations of commercial essential oils were systematically inhibited the pathogenic microorganism. The utilization of the particular commercially available essential oils, and their combinations with other components for preventing microbial resistance may be applicable.

## 3 Conclusion

Essential oils with known quality exert new biological and pharmacological activities that carry a utilization potential other than aromatherapy applications. Antimicrobial activity studies using essential oils and their major constituents targeting resistant bacteria may lead to the discovery of new drug leads with potential treatment of various microbial diseases. *P. nigra* and *T. serpyllum* oil combinations may have future potential as antimicrobial natural agents. The oil and antibiotic combinations offers a variation enabling relatively higher activity at lower concentrations. Also, combinations of major or minor constituents may not always responsible for synergistic activities, which should be evaluated carefully in depth. The effective combinations may form a basis for future research on upper respiratory tract infections as well as other microbial diseases.

However, more detailed studies, including *in vivo* experiments should be conducted. In addition further research must be performed to explore the use of combinations for the cosmetics and food industries with appropriate design.

## 4 Material and Methods

### 4.1 Material

*Thymus serpyllum* L. and *Pinus nigra* JF Arnold essential oils were obtained from a commercial source (Orlife Global International Trade Company, Istanbul, Türkiye). Essential oil constituents geraniol and α-pinene as well as the standard antibiotics tetracycline, amoxicillin, ciprofloxacin were acquired from Sigma-Aldrich Company (Munich, Germany) in 99% purity.

### 4.2 GC/FID and GC/MS Analysis

The GC-MS analysis was carried out with an Agilent 5975 GC-MSD system. Innowax FSC column (60 m x 0.25 mm, 0.25 μm film thickness) was used with helium as carrier gas (0.8 mL/min). GC oven temperature was kept at 60 °C for 10 min and programmed to 220 °C at a rate of 4 °C/min, and kept constant at 220 °C for 10 min and then programmed to 240 °C at a rate of 1 °C/min. Split ratio was adjusted at 40:1. The injector temperature was set at 250 °C. Mass spectra were recorded at 70 eV. Mass range was from *m*/*z* 35 to 450.

FID detector temperature was set to 300 °C. To obtain the same elution order with GC-MS, simultaneous autoinjection was applied on a duplicate of the same column applying the same operational conditions. Relative percentage (%) of the separated compounds were calculated from the FID chromatograms.

Identification of the essential oil components were carried out by comparison of their relative retention times (RRT) with those of authentic samples or by comparison of their relative retention index (RRI) to series of *n*-alkanes. Computer matching against commercial (Wiley GC/MS Library, MassFinder Software 4.0) and in-house “Başer Library of Essential Oil Constituents” built up by genuine compounds and components of known oils were performed.^41^

### 4.3 Antimicrobial Activity Test

The broth microdilution method was used to determine the Minimum Inhibitory Concentration (MIC) values of the essential oil, major constituent of essential oil, standard antibiotics, respectively.^42^ Essential oils concentration was prepared as 1 mg/mL, the standards were 8 μg/mL, and the major constituent was 10 μg/mL in the initial well. *Staphylococcus aureus* ATCC 6538, *Staphylococcus aureus* ATCC BAA44, *Bacillus cereus* NRRL B-3711, *Bacillus subtilis* NRRL B-4378, *Enterococcus faecalis* ATCC 29212, *Escherichia coli* NRLL B-3008, *Klebsiella pneumoniae* ATCC 4352, *Moraxella catarrhalis* ATCC 23245, *Salmonella typhimurium* ATCC 13311, *Staphylococcus epidermidis* ATCC 12228, *Streptococcus mitis* NCIMB 13770, and *Streptococcus mutans* ATCC 25125 were used as test bacteria. All microorganisms were adjusted to 1 × 10^8^ CFU/mL using McFarland No:5 standard in sterile saline (0.85%) solution. Essential oils were diluted and inoculated with strains. The microdilution plates were incubated at 37 °C for 24 h to allow bacterial growth. The resazurin solution (10%) (Sigma-Aldrich) was applied to confirm the values of MICs. This solution is used to detect bacterial viability visually based on a color change in microdilution assays. Each experiment was performed in duplicate. All test samples were dissolved in dimethyl sulfoxide (DMSO).

### 4.4 Checkerboard Microdilution Assay

The antimicrobial interaction between *T. serpyllum- P. nigra*, essential oil-antibiotics amoxicillin, ciprofloxacin and tetracycline (AMX, CIP, TET), and major constituents geraniol and α-pinene were tested using the checkerboard method. The initial concentrations were prepared as in the broth microdilution method in the 96-well plate. Subsequently, each well inoculated with tested bacteria suspensions (1 × 10^8^ CFU/mL) further cultivated at 37 °C for 24 h. Following this step, resazurin solution was used for the visual detection of MICs in the combination assays.

In the checkerboard assay, the fractional inhibitory concentration index (∑FICI) was determined by calculating the sum of the MIC fractions, each representing the MIC of test compound in combination divided by its MIC used alone. The associated effects were classified as follows; FICIs ≤ 0.5, synergism; FICIs 0.5 ≤ 1, additive effect; FICIs > 1–4, indifferent effect and FICIs ≥ 4, antagonism,^43^ respectively.

### 4.5 Toxicity Assay

The *in vitro* assay was performed using the MTT [3-(4, 5-dimethyl­thiazolyl-2)-2,5-diphenyl­tetrazolium bromide] assay on the HEK293 (Human embryonic kidney cells) cell line. The percentage of viable cells was quantitatively assessed through a colorimetric method as previously published.^44^ The cells were grown in DMEM supplemented with 10% FBS and 1% penicillin/streptomycin at 37 °C in a 5% CO_2_ atmosphere. HEK293 cells were treated with the combinations at concentrations where interactions were observed after 24 h. The optical absorbance was measured at 570 nm using an ELISA microplate reader (Thermo Multiscan Spectrum). The cell viability (%) was detected by using the following equation:

$$\mathrm{Viability}\%=\left(\left({\mathrm{Absorbance}}_{\mathrm{combination}}\right)/\left({\mathrm{Absorbance}}_{\mathrm{control}}\right)\right)\times 100$$

## Supplementary Material



## Notes

GC-MS chromatograms of the essential oils (PDF)
