# Supplementary material for: In Vitro Antibacterial Potential of Pinus nigra-Thymus serpyllum Essential Oil and Antibiotic Combinations
Source: ACS Omega. 2025 Dec 9;10(50):61528–34. doi: 10.1021/acsomega.5c07183 (PMC12750375; doi:10.1021/acsomega.5c07183)
Supplement: Supplementary file 1 [file ao5c07183_si_001.pdf]

In vitro antibacterial potential of *Pinus nigra-Thymus serpyllum* essential oil and antibiotic combinations

Sümeyye Elif Kahya[1*], Ayşe Esra Karadağ[1], Betül Demirci[2], Fatih Demirci[2*]

[1]Department of Pharmacognosy, School of Pharmacy, Istanbul Medipol University, 34820-Beykoz, Istanbul, TÜRKİYE

[2]Department of Pharmacognosy, Faculty of Pharmacy, Anadolu University, 26210-Eskişehir, TÜRKİYE

[*]Corresponding authors:

Fatih Demirci, Anadolu University; e-mails: fdemirci@anadolu.edu.tr, and demircif@gmail.com

Sümeyye Elif Kahya, Istanbul Medipol University; sumeyye.kahya@medipol.edu.tr

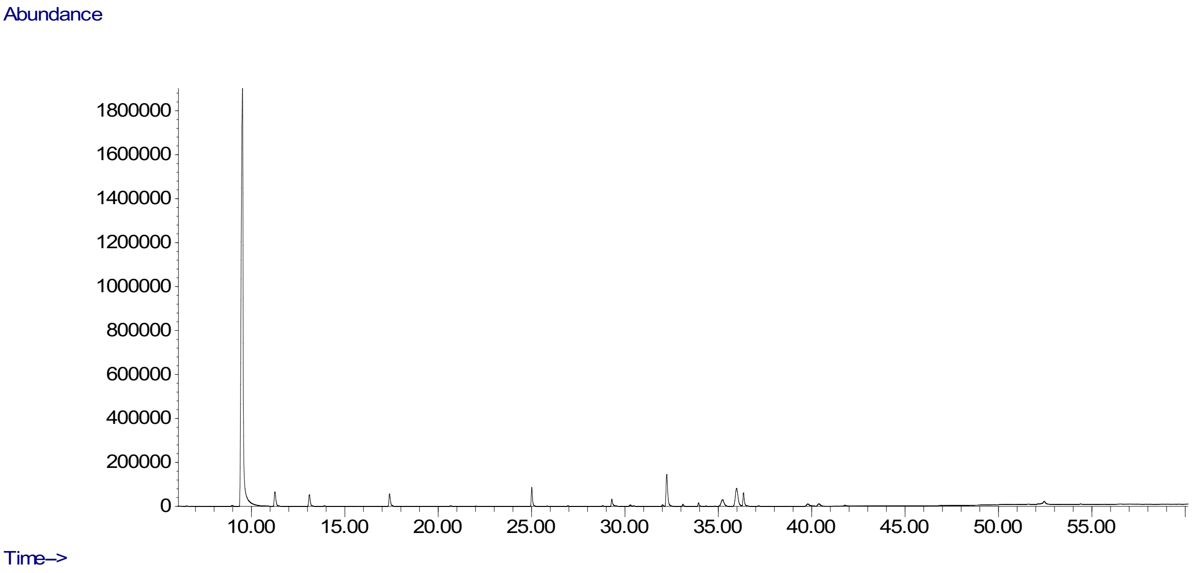

Figure S1. GC/MS Chromatogram of *P. nigra* essential oil

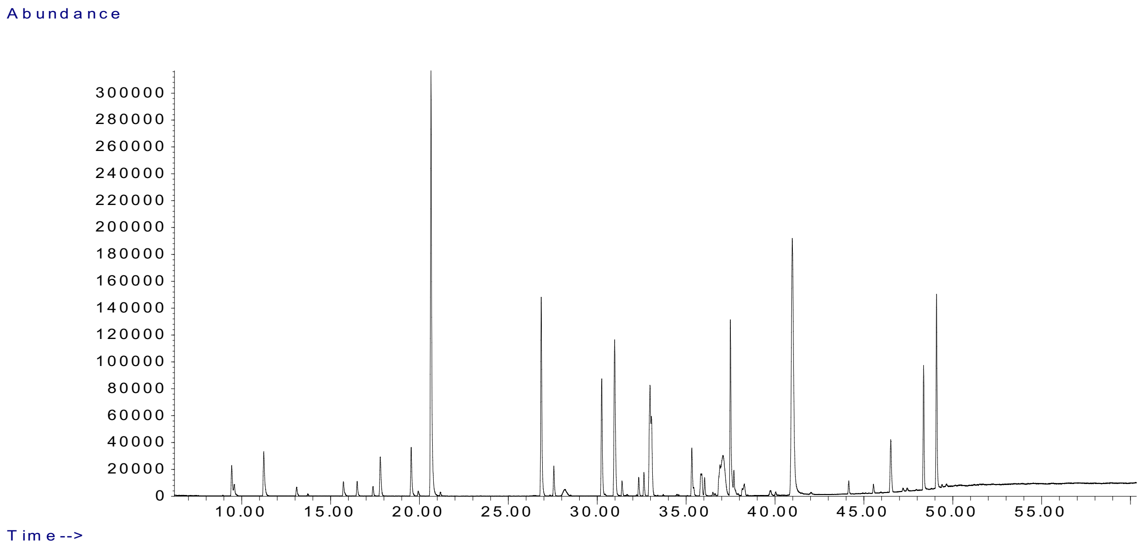

Figure S2. GC/MS Chromatogram of *T. serpyllum* essential oil
